# Initial clinical and health economic analysis of the GUIDE model

**DOI:** 10.1002/alz70858_104169

**Published:** 2025-12-26

**Authors:** Julius Bruch, Joel Salinas

**Affiliations:** ^1^ Isaac Health, New York, NY, USA

## Abstract

**Background:**

Dementia care often involves fragmented care pathways, resulting in increased healthcare utilization and costs. The CMS GUIDE (Guided Integrated Dementia Engagement) model aims to address these challenges by providing coordinated, evidence‐based care for dementia patients and their families. We present some of the first clinical and health economics results from an established track participant.

**Method:**

A retrospective analysis compared 85 participants enrolled in the GUIDE model with a matched cohort of 85 non‐participants based on age, gender, and dementia diagnosis. Healthcare utilization metrics, including emergency room visits, hospital admissions, and total hospital days, were assessed over two four‐month periods (pre‐ and post‐enrollment). A difference‐in‐differences approach evaluated changes in healthcare usage and costs, adjusted using standardized rates from published sources.

**Result:**

Participants in the GUIDE model demonstrated a 38% reduction in emergency room visits and a 28% decrease in hospital days compared to a 7% reduction and a 4% increase, respectively, in the comparison group. Ambulatory care visits increased by 10% for participants, aligning with the model's focus on proactive care. Total cost of care (PMPM) decreased by 22% for participants compared to a 2% increase in the comparison group. These findings suggest a meaningful shift toward reduced acute care utilization and improved care delivery within the GUIDE model.

**Conclusion:**

The GUIDE model shows promising potential in reducing acute care dependency and fostering cost‐efficient dementia care pathways. While initial results highlight its effectiveness, further studies over extended timeframes are essential to validate its long‐term clinical and economic impact.

